# Bloody technology: the sphygmograph in asylum practice

**DOI:** 10.1177/0957154X17700292

**Published:** 2017-03-31

**Authors:** Jennifer Wallis

**Affiliations:** Queen Mary University of London, UK

**Keywords:** Blood, medical technologies, pharmacology, physiology, sphygmograph

## Abstract

The sphygmograph, an instrument to measure and visually chart the pulse, was used by a number of asylum researchers in the late nineteenth century in an attempt to better understand mental disease. In charting the use of such a medical technology in the asylum, this article explores the utility of a practice-oriented approach in the history of psychiatry – as a window onto the alienist profession and as a means of investigating how new medical technologies were assimilated into everyday practice.

## Introduction

In 1889 Herbert Hayes Newington, President of the Medico-Psychological Association, considered ‘a fact that we all admit, nay, that we are all fighting for, which is that insanity is primarily and essentially an expression of disease of the body’ (Newington, 1889: 300). The idea that mental disease could be explained by structural anomalies within the body gained ground in the latter part of the nineteenth century, evidenced in the increasing interest of asylum doctors in pathological and physiological research. To those who believed that ‘insanity had no pathology’, one could counter that this was merely a consequence of ‘present imperfect means of investigation’ (Greenlees, 1887: 472). In the drive to make the interior state of the body visible, new medical technologies and graphical methods were central in gaining access to psychophysiological phenomena. From the mid-nineteenth century, a range of registration apparatus was available that held out the promise of redrawing the boundaries of medical expertise and, with it, transforming contemporary understandings of disease and the body. Much of this new technology was concerned with processes within the body that lent themselves to graphical or statistical narratives. Temperature charts were a simple form of this. William Bevan Lewis’s ‘On the thermal changes in epilepsy and the epileptic status’ (1876), for example, correlated temperature and seizures. Here, a rise in temperature after a seizure was interpreted by Bevan Lewis as indicating ‘a running off of nervous energy of a low type’, linking physiological changes to unseen mental processes (p. 300).

In such articles, however, the reader is often left to fill in the gaps between the initial impetus for investigation and the conclusions drawn. The marks on the chart – like the ‘time curves’ of Hermann von Helmholtz – may be ‘small and exquisite’, but they represent something much larger (Schmidgen, 2014: 45). The temperature chart omits the work behind its production: the choice and acquisition of thermometers, the interaction between doctor and patient as the temperature is taken, the working of the instrument, the recording of the result, and the reproduction of this result for a published article. Warner (1995: 182) notes that as well as what doctors said, there is also a need to pay attention to what they did. This exhortation was echoed more recently by Worboys (2011: 109) when he wrote that ‘historians of medicine still have much to learn from the “practice turn” in the history of science’. His emphasis on the place of practice is an important one. Practice theory describes the attempt to move beyond an emphasis on grand structures as the defining elements in science; it grants agency not only to the doctor or the hospital, but to smaller-scale, everyday aspects of scientific work. Within practice theory, we can more easily conceive of science as a multi-authored, multi-faceted activity. These multiple practices involve processes of resistance and accommodation – what has been termed ‘the mangle of practice’ (Pickering, 1995: xi) in which, for example, a piece of equipment may behave differently than expected and force a reconsideration of theory. It is an approach in which objects and subjects of scientific knowledge are capable of influencing facts and observations.

Practice theory has clear relevance to histories of medicine and psychiatry, particularly when the issue at hand involves the physical or pathological investigation of the body. Despite Newington’s emphasis on the centrality of the body to nineteenth-century conceptions of mental disease, bodies have been much less present in the history of psychiatry than in the history of medicine more broadly. On the surface they appear to loom large, subjected to shower baths, electrical current, or ice picks to the brain, but these are often accounts in which the body is defined by apparently ‘barbaric’ treatments and consequently ‘re-Othered’ in the historical telling (Dreger, 2007). Also, within demographic and statistical analyses of asylum populations, the individual patient becomes difficult to see except as one part of an amorphous mass that is counted and charted in what can often become a modern-day reflection of the nineteenth-century classificatory impulse. This relative absence of the individual body from the history of psychiatry is especially striking when the nineteenth century is under discussion, a point in time when there was ‘a strong desire … to construct a coherent narrative of the body’ (Coleborne, 2007: 170). Constructing such narratives depended to a significant extent on existing medical techniques and technologies, and here again the historian is forced to confront the issue of objectification. Although there has recently been a shift away from the notion of a ‘socially-constructed’ body that is examined, talked and thought into submission, the notion of medical technologies as instruments of professional power is a difficult one to move past. In seeking to reintegrate practices into the history of psychiatry, it is rather easy to erase the patient inadvertently; in works such as Andrew Scull’s *Madhouse* (2005), which provides a gripping account of the focal sepsis theory and its associated practices, the patient appears to shrink somewhat beneath the surgeon’s knife.^1^

In focusing on the psychiatric patient as a body of knowledge bound up with particular practices, then, it becomes necessary for the historian to address these broader issues of construction or mediation: in examining the body and medical technologies in the history of psychiatry, are we simply ‘objectifying’ the patient? Joel Snyder (1998: 381) has described machinic inscriptions of bodily processes as additions to the human body that can never really ‘erase’ the patient by virtue of the fact that they cannot fully capture them. Going further, I would suggest that by focusing on the everyday practices and technologies brought to bear on the body, we might pull it (and the patient) more directly into the narrative. The means by which a disease comes to be ‘known’ rely on the doctor, patient and multiple processes that are affected by the actions of both, as well as by medical technologies. Wailoo (1997: vii) has eloquently described how blood technologies have ‘become props for working out larger problems of disease identity, patient identity, and professional identity’. These wider-ranging implications of medical technology are what the present article seeks to investigate. In viewing the graphical traces of a technology (such as the temperature chart) as an end point that somehow erases the patient at their heart, we also risk erasing the scientific work that produced the trace itself. The ‘mangle of practice’ is a crucial part of the story of any medical technology. In pursuing the history of a medical technology, we are able to pursue something more than the life of the technology itself: interactions between doctors and patients, the organization of medical practice, and the dissemination of specialist or technical knowledge.

## Bloody technologies

In the nineteenth century, the desire to ‘coax narratives of madness from blood’ (Noll, 2006: 396) manifested itself in microscopical and serological investigation, beginning with William Lauder Lindsay’s laboratory examination of the blood of asylum patients in 1854 (Noll, 2006: 399). As a substance flowing through the body and touching upon all organs, blood was a charismatic scientific object that captured the imagination of researchers keen to investigate the body–mind relationship. This was evident in the physical examination of patients as well as in post-mortem investigation. Bevan Lewis (1889: 288) noted that the blood of several general paralytic patients was hard to collect on account of its sluggish flow, and several of his contemporaries posited a link between heart disease and insanity based upon their post-mortem observations (e.g. Burman, 1873a; Reynolds, 1896). Upon finding arterial degeneration and hypertrophy of the heart in several asylum post-mortems, T. Duncan Greenlees of Garlands Asylum offered a simple explanation for mental disease: the heart was unable to work to its fullest capacity and the brain received less nourishment as a result (Greenlees, 1885: 344). The suggestion that the organs of the body depended on a healthy blood supply raised the possibility that mental diseases were simply the consequence of impaired circulation. Indeed, a reviewer of the 1874 volume of the *West Riding Lunatic Asylum Medical Reports* suggested that recent investigation in asylum practice had been refined too far, overlooking common-sense physiological explanations: ‘It has been a fashion with some inquirers to fix their attention exclusively on the brain-cells … but on the rise and fall of the circulation the healthy action of the [brain] must depend’ (Anon., 1875a: 278).

In investigating blood flow, the pulse was a simple means of ‘seiz[ing] upon the essence of the closed, coded body’ (Stowe, 1996: 60). Early instruments for measuring the pulse and blood pressure were devices confined to the physiological laboratory, as they relied on an open artery to produce a usable inscription; the kymograph (or kymographion) intrusively measured blood pressure and inscribed the movements of the stylus on a circular drum of smoked paper. Developments in the nineteenth century overcame this difficulty, although the first of these were also intended for use in an experimental, rather than clinical, context. Karl Vierordt’s sphygmograph, though it could be applied to unbroken skin, was an unwieldy 168 cm long (Lawrence, 1978: 197). The turning-point was Étienne-Jules Marey’s much smaller sphygmograph of 1860, consisting of ‘a lever, one end resting on a pulsating artery and the other connected to a pen. A clockwork mechanism moved a strip of smoked paper under the pen at uniform speed, converting the pulsations into a pictorial form’ (Reiser, 1978: 101). Each curve on the tracing corresponded to a pulsation. Marey’s device did not excite much comment when it was introduced to Britain. A short piece in *The Lancet* in April 1860 was cautious about the device’s utility: ‘It can only indicate the frequency or the more or less regularity of the pulse. It may be doubted whether these instruments, though very ingenious, will ever actually prove useful in practice’ (Anon., 1860: 435).

Marey’s device was modified by a number of people who offered up their own variants of the apparatus, including the addition of a microphone (Dudgeon, 1882: 27) and weights that altered the pressure exerted on the wrist (Foster, 1868: 64). A significant development was the ‘pocket sphygmograph’ of Robert Dudgeon that, as its name suggested, was a less cumbersome device suited to the physician on his rounds. Despite the laudable aim of providing an objective pulse reading, untainted by the human experimenter, each modification of the instrument might produce a different result and be further affected by the technique of the person employing it. It was typically used to perfect or confirm a diagnosis already made by traditional percussion and auscultation (e.g. see Foster, 1866: 330), but – as a tool derived from experimental physiology – it was difficult not to be seduced by the charms of an instrument that ‘decompose[d] bodily motion into the smallest temporal and spatial segments possible’ (Douard, 1995: 186). This ‘wordless science’ (Daston and Galison, 1992: 81) had didactic use too, with the traces it produced capable of being printed in journal articles or distributed to colleagues.

We should not assume, however, that this was an instrument which easily found its way into everyday practice across Britain. In many discussions of the sphygmograph, the necessity for its handler to be an accomplished investigator was forcefully emphasized, as skills became ‘packaged’ into the instrument (Gooding, 1992: 74). Even in the first years of the twentieth century, it was described as a piece of apparatus that ‘need[ed] as many dexterities as a cricket bat’ (Anon., 1904: 560). Just as one could welcome the sphygmograph as a valuable tool, its mass manufacture meant that it could be used by almost anyone with the funds to obtain it but not necessarily in possession of the requisite specialist skills. ‘No one’, wrote one of the instrument’s advocates, ‘should attempt to use it who cannot readily obtain exactly similar tracings from the two radials of a healthy person’ (Mahomed, 1873: 141). It is notable, then, that detailed accounts of the sphygmograph’s experimental use and clinical applications were produced by medical men in asylums, institutions generally perceived to be beyond the boundaries of mainstream scientific or physiological research. Yet, as Eric Engstrom’s work on German psychiatry has shown, a wide range of instruments and techniques were employed by asylum doctors in the nineteenth century in the hope of ‘elicit[ing] new signs and symptoms of madness’ (Engstrom, 2003: 130).

George Thompson, a Clinical Clerk and Medical Officer at the West Riding Asylum, and later Superintendent of Bristol Asylum, produced three articles on the use of the sphygmograph in the early 1870s. The first, ‘The sphygmograph in lunatic asylum practice’ (Thompson, 1871), conceived of the instrument as ‘an impartial and consistent witness’ (p. 59) in the study of the pulse and as a means of rectifying the time ‘wasted’ on microscopical examination of the brain substance (p. 58). This reaction against the microscope was a theme that Thompson returned to in a later article: although the microscope ‘may enable us to make out “disseminated molecular degeneration,” “proliferation of cellular tissue,” or “fatty decay”’, he asked whether this ‘carr[ied] us any farther?’ (Thompson, 1875: 580). The sphygmograph was intriguingly situated by him, then, as representing modern scientific practice, yet at the same time as something of a reversion to simpler physiological investigation in the face of cynicism about microscopical methods. Thompson employed the instrument enthusiastically: in his 1871 article and in ‘The sphygmograph in epilepsy’ (Thompson, 1872), he presented several sphygmograph tracings alongside case histories to demonstrate states of health and excitement, as well as fits. (In a later article, ‘On the physiology of general paralysis of the insane, and of epilepsy’, Thompson (1875: 579) expressed regret that these two articles had been relatively brief, suggesting they had been heavily edited.) The sphygmograph was also discussed in some detail by Bevan Lewis (1881) and Greenlees (1885, 1887). In applying the sphygmograph to the study of mental disease, asylum researchers demonstrated their growing commitment to the notion of mental disease as bodily disease, as well as their willingness to draw upon the practices of other fields of contemporary science and medicine.

## The use of technology in the asylum

The sphygmograph frequently appears in the casebook records of both the West Riding and Bristol Asylums of the late 1860s and 1870s. Its appearance coincides with Thompson’s time at both asylums (and the appointment of J. Wilkie Burman at the West Riding) and continues, albeit less frequently, into the 1880s. While some tracings were taken from patients soon after admission – such as that of George D., admitted to the West Riding on 9 December 1880 and tested with the instrument five days later^2^ – it does not appear to have been a routine admission examination. Rather, it was an examination appearing in clusters, presumably when a Medical Officer had time to undertake it alongside his other duties.

As an instrument measuring something as minute as the pulse, the sphygmograph required a good deal of care in its use to obtain an adequate pulse tracing. Marey’s model fastened the instrument to the arm with a lace bandage, but the careful positioning of the arm also had to be considered, as described in Arthur Ernest Sansom’s *Manual of the Physical Diagnosis of the Diseases of the Heart* (1881), where he dedicated two full pages to the correct position of the arm and set-up of the equipment (pp. 230–1). When dealing with patients suffering from mental distress or neurological complaints, however, there were further considerations. The difficulty of performing any kind of physical exam is evident in many asylum records: of a patient at Bristol it was noted that ‘stethoscopic examination [could not] be made on account of muscular tremor’.^3^ In his attempts to apply the sphygmograph to Garlands Asylum patients in the last stages of general paralysis of the insane (GPI), Greenlees (1887: 478) obtained a tracing from one only after ‘many unsuccessful attempts’. In several cases of epilepsy, he resorted to taking tracings while patients were unconscious (p. 475).

A common obstacle when seeking to apply the sphygmograph was persuading the patient of the harmless nature of the activity. Indeed, simple auscultation often proved difficult before such an elaborate instrument as the sphygmograph had been introduced; at the West Riding it was recorded for a female patient that ‘[a]uscultation could not be satisfactorily performed as she makes persistent attempts to lay hold of the stethoscope imagining that its application would hurt her’.^4^ Some patients refused to cooperate with the examination and, it seems, triumphed over the doctor. A patient of G. Hunter Mackenzie at Gloucester Asylum ‘persistently refused the application of the sphygmograph’ (Mackenzie, 1878: 248). This was often due to fear of the instrument; Edinburgh Royal Asylum physician Lewis Bruce related that in one case ‘it was almost impossible to obtain a tracing, as the patient regarded the sphygmograph with horror and fear’ (Bruce, 1895: 63). Certainly, some doctors recognized that the use of instruments entailed anxiety for the patient, and they offered advice such as: ‘Guard against all cases of transient excitement, especially as regards the instrument. Put the patient quite at ease by letting him see it applied to others, or by taking one or two preliminary tracings of himself’ (Mahomed, 1873: 141).

If the heart was not explicitly investigated as ‘an organ of *emotion*’ at this time (Bound Alberti, 2010: 87, original italics), there is nevertheless a sense in the research discussed here that a patient’s emotional state could alter the character of the circulation. In *The Asclepiad* of 1888, Benjamin Ward Richardson described the mind as a ‘stimulant’ to the pulse, with particularly marked changes taking place ‘in persons of a very observant nature or of a peculiarly sensitive turn of mind’ (Richardson, 1888: 238). Burman addressed the issue directly in ‘Heart disease and insanity’ (1873a), where he acknowledged the role of apprehension during physical examination and advised taking two tracings (one when any excitement had subsided), but others argued that such physical examination was inappropriate, however calm the attending doctor judged the patient to be. Thomson (1890: 155), in a letter to the *Journal of Mental Science*, criticized the extension of ‘the cold-blooded examination of an ordinary hospital patient’ to the patient resident in an asylum. Alluding to the delusions held by patients and the effect of subjecting them to examination with unfamiliar and frightening instruments, Thomson could ‘just fancy a case of acute mania or melancholia being percussed, auscultated, the sphygmograph, thermometer, plethysmograph etc., used upon him’ (p. 155).

Despite such reservations, the sphygmograph continued to be applied to a number of asylum patients in the study of the physical bases of mental disease. Although it was applied to almost every form of mental disease, general paralytic and epileptic patients presented the most interesting cases. The sphygmograph offered to chart the bodily deterioration of GPI, but also the abrupt somatic manifestations of epilepsy, if one was able to employ it before a fit came on. Pulse tracings could provide a vivid account of physical events. One of the tracings reproduced by Greenlees (1887: 483) is striking in its inscription of the event of death itself, captioned ‘Dying from a series of Epileptic fits’. Here, the instrument takes on an almost supernatural quality, illustrating ‘the drama of the heart for Victorian physicians’ (Kennedy, 2014: 116). Patients with relatively rare diagnoses also provoked interest: in the 1880s a male West Riding patient diagnosed with locomotor ataxia had three tracings taken before being discharged.^5^ Further, the sphygmograph was used to investigate the physiological changes that occurred during hypnosis (Anon., 1892; Tuke, 1883), and in a case of ‘dual brain action’ (Bruce, 1895). Bruce’s patient appeared to have ‘two separate and distinct states of consciousness’ (p. 54) which were differentiated by the language spoken (English or Welsh). Bruce also claimed that these two states produced different sphygmographic tracings (p. 63).

In examining the results of sphygmographic investigation, several doctors commented that the character of the pulse resembled the character of the mental disease. Thus, patients with mania had a predictably fast pulse, resembling that seen in fever or acute disease; those with melancholia had a slower pulse; and in epilepsy the pulse was quick and ‘wanting in tone’ (Greenlees, 1887: 473–5). Researchers like Greenlees attempted to embed the sphygmograph in asylum practice as an instrument able to discern mental as well as physical anomalies via the measurement of bodily phenomena. In GPI, the expectation that almost every part of the body would be affected made the condition a natural starting point when investigating the circulation in mental disease. The pulse of GPI patients also appeared to alter according to the stage of disease; Greenlees observed that towards the end of the second stage of the condition, one could discern a ‘plateau-like summit to the tracing … indicat[ing] a high tension of the pulse, sustaining the lever of the instrument for some time before the emptying of the vessels permits of its fall’ (p. 477). Here we can see how a graphical instrument could produce diagnostic phenomena visible to the naked eye (the sustained movement of the lever) that complemented the final tracing. Bevan Lewis noted how one could already – before the tracing was obtained – make pronouncements on the character of the pulse: as GPI advanced, more pressure was needed on the artery to obtain a satisfactory tracing, this low occlusion pressure indicating ‘enfeebled muscle’ (Bevan Lewis, 1881: 4).

In ‘The sphygmograph in lunatic asylum practice’, Thompson (1871) presented several sphygmographic tracings, but explicitly compared two to elucidate the nature of GPI. One of these tracings came from his own research, the other from W.B. Carpenter’s *Principles of Human Physiology* (1869); the two bore a remarkable resemblance in their ‘slanting and short’ lines of ascent and ‘gradual and prolonged’ lines of descent (Thompson, 1871: 63–4). While Thompson’s example represented the pulse of a general paralytic patient, Carpenter’s illustrated the pulse ‘of a healthy individual who had been immersed in cold water for some length of time and who was … in a state of chill’ (p. 64). To Thompson, it was evident that in both cases a spasm of the vessels was being recorded. However, his faith in his work did not convince the reviewer of the volume of the *West Riding Medical Reports* in which his piece appeared. ‘[W]e must say that we should not care to trust to the sphygmograph alone for the diagnosis of general paralysis’, they wrote, expressing concern that the device’s readings should not be blindly accepted (Anon., 1872: 560). There was a sense that Thompson had misunderstood the point of the sphygmograph. Physiologist John Burdon Sanderson, a keen user of the instrument, saw it as something complementary to the physician’s observations and a possible means of picking up indications of fatal or chronic conditions. He did not recommend using it to investigate mental diseases or transient phenomena such as fits, and even in accounts written by advocates of the instrument it comes across as a somewhat untrustworthy piece of equipment. In his *Handbook of the Sphygmograph*, John Burdon Sanderson (1867) made clear that the instrument was ‘not to be regarded … as an aid in the discovery and discrimination of organic diseases … Its use [was] to enable the physician to investigate the state of the circulation … in diseases of which the general nature is already recognised’ (p. 27). The sphygmograph could also be a valuable means of demonstration, teaching medical students about the characteristics of the pulse in a mechanical language rather than relying on the verbal descriptions of a human demonstrator (p. 66).

Notwithstanding the warnings of Burdon Sanderson, the tracings of the sphygmograph were accorded significant attention in several published articles by asylum doctors, both as proof of the author’s arguments and as visual aids to understanding. It is noteworthy that discussions of a tracing’s significance are less evident in original casebook records. Thompson’s confidence in his tracings was suggested by reference to a tracing that ‘confirmed’ a diagnosis of GPI (Thompson, 1871: 65). The tracing had attracted no special attention in the original casebook record, however, suggesting that sphygmographic inscriptions were, despite their surface objectivity, part of a shifting narrative of disease, one variable of which might be the retrospective gloss of the researcher.^6^ Even if one accepted that a tracing was the result of ‘objective’ mechanical recording, then, it was not immune from being read in various ways after its creation. Further, despite the apparent difficulties of using the instrument, there is little mention of the procedure itself in asylum casebooks. Thompson (1872: 304) assures the reader that ‘[m]uch care was taken in avoiding any undue excitement while the sphygmograph was being applied’, but he does not explain how exactly this was carried out, or whether it was effective. In one case, the date of the sphygmograph tracing in the casebook (scratched onto the corner of the smoked paper along with the patient’s name) corresponds with a dated written note that omits mention of the procedure entirely, instead noting the patient’s bowel movements.^7^ The tracing, then, might be seen to stand as a narrative in and of itself that did not require any further explanation. Conversely, it may not have been viewed as an end point, but as an artefact to be further processed for publication or other dissemination. It is also possible that those doctors carrying out sphygmographic tests were not the doctors who were writing casebook records.

Tracings were evidently valuable enough as medical objects for attention to be devoted to their physical characteristics: Thompson described producing positive prints that could be pasted into books or distributed to colleagues (Anon., 1877). In an interesting case record at Bristol, a Medical Officer drew by hand a section of a sphygmographic tracing, presumably copying from the original (not present in the record); his inked inscription is accompanied by a written description of the pulse.^8^ This artfully-rendered version of the tracing recalls what Brain (2015: xviii) terms the ‘hybrid ontological status of [the physiological laboratory’s] scientific objects, suspended between natural facticity and artificial creation’. The use of mechanical instrumentation did not necessarily replace other forms of expression. Indeed, despite enthusiasm for mechanical visual renderings of the pulse, other narratives could be equally evocative and informative. A Bristol Medical Officer described the pulse of a patient as ‘very rapid, small and thready’;^9^ another wrote of the heart’s ‘first sound [as] loud & whizzing’^10^ – observations conjuring up vivid sensations that graphical pulse tracings could not fully capture. Signs of circulatory problems could also be garnered from simple physical examination, such as cold extremities: ‘Hands & feet have a purplish hue & circulation in them [is] evidently sluggish.’^11^ The sphygmograph was one ‘way of knowing’ disease (Pickstone, 2000) that could be used alongside other methods in assessing a patient’s condition, though it seems that some researchers (such as Thompson) placed more faith in it than others, and appealed to its data in relative isolation from other factors.

## The sphygmograph and drug treatments

The varied responses to, and uses of, the sphygmograph raise the question of how far the instrument was simply an example of ‘data rather than the patient [claiming] the physician’s attention’ (Borell, 1993: 259). There is, I would argue, a little more to the story, as the sphygmograph became implicated in therapeutic interventions in the asylum, particularly in cases of GPI. As Evans (1993: 789) has emphasized, the adoption of the sphygmograph in clinical as well as physiological contexts saw the instrument’s functions alter, as the knowledge it produced was applied to new questions. In the West Riding and Bristol Asylums, pharmaceutical interventions were introduced that aimed to act upon vessel anomalies apparently identified by the sphygmograph. The high arterial tension described by Bevan Lewis and Greenlees was frequently compared to Bright’s disease, with doctors commenting on the similar tracings obtained in both conditions (e.g. see Bevan Lewis, 1881: 3). Indeed, high tension was observed in enough asylum patients for Greenlees (1887: 479) to come to the conclusion that ‘increased arterial tension [was] the rule in cases of mental defect’. The similarity between GPI and Bright’s disease raised the possibility that both were dependent on some form of toxin, and the frequent implication of alcohol in GPI appeared to confirm this. The alcohol question was one that would be addressed in more detail as serological investigations in the later nineteenth and early twentieth centuries focused attention on the quality of the blood rather than its quantity or flow. More immediately interesting were the treatments indicated by high arterial tension or spasm, and the regulation of blood flow to the brain; an unusual response was that advocated by T. Claye Shaw of St Bartholomew’s Hospital, who appealed to sphygmography in his advocacy of trephining (Shaw, 1889: 1090).

Shaw’s surgical response was an extreme one that did not go uncriticized by his contemporaries (see Revington, 1890); in terms of treatment, the late nineteenth-century asylum was more often characterized by pharmaceutical intervention. That this was a significant undertaking is suggested by annual reports detailing physical alterations to asylums to provide specific sites for pharmacological activities; at the West Riding in 1870, a disused cellar was converted into ‘a Laboratory for the Preparation of Drugs’.^12^ Drugs also occupied other spaces in the asylum, and were investigated in laboratories and tested on the wards. Digitalis was investigated at Gloucester asylum (Mackenzie, 1878), ergot at the West Riding (Churchill Fox, 1871), and ergot, belladonna, and subcutaneous morphine injection at Bristol.^13^ The drug that became associated with sphygmography and the modification of the circulation was Calabar bean. Calabar (*Physostigma venenosum*) is a poisonous seed from an African plant, which was brought to the West in the nineteenth century by returning missionaries. Though lethal if ingested in anything more than minute quantities, it was put to a number of uses in the late nineteenth century due to its ability to paralyse the muscles – it calmed the spasms of tetanus and proved to be the first effective treatment for glaucoma when applied directly to the eye. As a drug that had the effect of paralysing the muscles, Calabar was of interest in the asylum due to its ability to ‘[dilate] the peripheral vessels, as well as markedly [depress] the heart’s action; … [thus] lower[ing] the blood pressure on the brain’ (Fothergill, 1874: 78), as well as potentially calming seizures. The extract was administered to patients in liquid form.

Thompson’s 1871 article was almost evangelical in its praise of Calabar, suggesting that it was a treatment with the staunch support of West Riding Superintendent James Crichton-Browne; it ‘had been recommended by him as a valuable means of exercising a favorable influence on the course of [general paralysis]. In every case, its employment has been attended with marked benefit’ (Thompson, 1871: 67). Thompson related the case of W.V., a 35-year-old man admitted in 1871: ‘[The patient was] received … with undoubted symptoms of general paralysis. One sixth of a grain of the Ex[tract]. physostig. was ordered, and has been continued up to the present time with marked improvement’ (p. 68). Similarly, the tone of his report on S.M. suggested that his symptoms had been lessened by the use of Calabar:

When admitted to the West Riding Asylum he had exalted ideas; there were inequality of the pupils, tremor of the lips, and awkwardness of gait. … [After three months of treatment with Calabar bean] there was such marked improvement in his condition that the use of the extract was discontinued. (p. 68)

Throughout the 1870s Calabar was administered to West Riding patients soon after admission, if a diagnosis of GPI was made or suspected. Thomas G., admitted on 4 July 1873, was prescribed Calabar on 24 July, the date on which it was noticed a haematoma of the ear was commencing.^14^ His dose was increased in October, though he remained very excitable, suggesting that the impact of the extract on his symptoms was limited. Although James Sherlock at Worcester Asylum credited the drug with bringing about ‘sufficient improvement [in two general paralytic patients] … to enable them to return home to the care of their friends’ (Anon., 1873: 455), the chronic nature of GPI meant that Calabar could hardly be construed as a ‘cure’ in the true sense of the word. Indeed, Crichton-Browne felt compelled to write to the *Journal of Mental Science* to make clear that he did not, as Thompson seemed to have suggested, view Calabar as a potential end to GPI (Crichton-Browne, 1875). Slowing the progress of the condition was, he said, the most that could be done. Burman described a patient ‘whose chief delusion [was] that he [was] possessed of 10,000,000 tons of coals – a delusion which thanks to the continued use of the Calabar Bean, he has been enabled to enjoy the pleasure of for several years’ (Burman, 1873b: 540). This anecdote raises the issue of the quality of life for such patients. Several commentators pointed out the risks attendant upon Calabar’s use – risks that they claimed Thompson had purposefully understated. As one doctor interpreted Thompson’s 1875 piece, the action of Calabar was in reducing the action of the heart to such an extent that it had ‘produce[d] fainting in one case, while the patient was out for a walk’ (Anon., 1875b: 150). Elsewhere, the use of the drug had been linked to asphyxia (Fraser, 1867: 323). Such concerns are rarely alluded to in casebook records, although it is clear that Calabar was viewed as an unsuitable treatment for patients experiencing prolonged periods of excitement. In several instances, the drug was withdrawn for this reason: after taking an eighth of a grain twice a day, one patient was said to be ‘much excited [and] the calabar bean [was] to be discontinued for a few days’.^15^ Thompson (1875: 586) stated that he used the extract until ‘excitement and occasionally an epileptiform seizure [had] been brought on’, before stopping its use. He was still employing Calabar in the mid-1880s during his time at Bristol.

During treatment with Calabar and other drugs, the sphygmograph was used to chart the effect of medication on the circulation. Bevan Lewis (1881) used it to investigate the impact of digitalis on the body, and it was an instrument that complemented pharmacological interventions as a quick and direct means of assessing their effects. At Bristol in 1874 a patient had their Calabar treatment immediately stopped when a pulse tracing was taken that was thought to demonstrate ‘cardiac hypertrophy with easily dilated arteries’ (a striking illustration of the faith placed in the detail to be extracted from the instrument).^16^ The sphygmograph could also be employed in a more experimental context. The record of a West Riding patient in the early 1880s contains two tracings, one depicting the throes of a maniacal attack without the influence of any drug, the other after an eighth of a grain of hyoscyamine.^17^ The character of the two is quite different, the first displaying steep and high arcs, the second being much flatter. Thus, the sphygmograph continued to be used in a clinical-therapeutic context to assess the effect of new drug treatments on the physical and mental state of patients, as well as a means of investigating the characteristic features of different types of mental disease.

## Conclusions

During a discussion at the Medical Society of London in 1889, one participant dismissed the sphygmograph as a device of ‘little use except as a recording instrument’ (Anon., 1889: 1337). Many histories of blood technologies agree. Lawrence (1978: 199) describes the instrument as ‘the passion of a few specialists’ who had a tendency to overestimate its potential. The sphygmograph was not a device that was widely adopted in clinical practice (Romano, 2002: 89). That the sphygmograph was not a success story, however, does not mean that there is nothing to be learned from studying its use. As an instrument typically associated with experimental physiology, it is notable that detailed accounts of its use come from asylums, institutions generally viewed as somewhat removed from ‘mainstream’ contemporary medicine. Lawrence (1985: 517) asks if instruments at this time were ‘a sort of Trojan horse by which a new breed of medical man was attempting to show the value of the new basic sciences to bedside medicine’. The use of the sphygmograph in the West Riding and Bristol Asylums suggests that it was an instrument with the potential to link the bedside and the bench more closely, with physiological examination feeding into therapeutic endeavours. Its use as investigated in this article also highlights the multiple roles that an asylum doctor might perform – physiological investigator, psychiatric assessor, drug dispenser, and writer and researcher. Doctors’ introduction of instruments like the sphygmograph into an asylum context appears in some cases to have been a personal endeavour, not necessarily linked to a broader research project, but borne out of a desire to apply the methods of other fields to psychiatry in the drive to comprehend mental disease. Thus, we can see how ‘[t]he history of technical work in hospitals [and, here, the asylum] presents an opportunity to disrupt notions of tightly contained occupational groups’ (Twohig, 2006: 74). This is particularly relevant when the late nineteenth century is under discussion – a period when the boundaries between medical specialties were still evolving and subject to debate.

The sphygmograph was not an instrument that was employed without a consideration of its technical limitations – indeed, the difficulty of reading ‘a language that medicine was only beginning to understand’ (Frank, 1988: 274) was explicitly addressed by several researchers. Although it may have held out the promise of connecting the bedside and the bench, it was not necessarily a straightforward way of doing so, as Burdon Sanderson (1867: 65) recognized: ‘Let us … avoid being in too great a hurry to introduce the sphygmograph into the consulting-room; for if, with so imperfect a knowledge as we at present possess of first principles in diagnosis, we shall not only discredit ourselves, but the method by which we profess to be guided.’ The sphygmographic tracing itself was a partial picture, something that only made complete sense with knowledge of the machine that had drawn it (Schmidgen, 2014: 27–8). As a ‘short, incomplete, and disconnected’ record (Vaughan, 1888: 1379), the tracing became even more problematic when the phenomenon under investigation was itself transient – such as the epileptic fit or maniacal attack. Although Thompson might have imagined the sphygmograph as an ‘impartial witness’ (Thompson, 1871: 59), self-registering technology did not ‘[make] it possible to separate the act of receiving medical data from the act of interpreting it’ (Borell, 1993: 267). It is important to emphasize that the sphygmograph was generally an instrument used not in isolation, but alongside regular physical examination. The suggestion that certain forms of mental disease were associated with vessel anomalies was one that relied on simple visual or sensory assessment (cold extremities) and post-mortem exam (congested or deteriorated vessels), as well as the tracings produced by the sphygmograph. Towards the end of the century, the sphygmograph was also used alongside other blood technologies, such as Gower’s haemocytometer and the haemoglobinometer, part of an increasing ‘technicalization’ of the asylum (e.g. see Bevan Lewis, 1889: 161). The fact that researchers such as Thompson placed such emphasis on the sphygmograph, without reference to other forms of evidence, might suggest that this was a group peculiarly seduced by the instrument’s potential to bestow some kind of professional status, or that the instrument’s traces served more as symbols of a particular way of working than as accurate representations of physical phenomena (see Schmidgen, 2014: 175). However, we should also recognize that medical technologies were not used by all researchers in a uniform way; as individual historical actors, doctors framed instruments with regard to their own research interests, personal theories and everyday practices.

In the Introduction to this article, I suggested that taking a practice-oriented approach was a way to bring together histories of the body with the history of psychiatry – a combination particularly relevant to the late nineteenth century, as asylum doctors sought a somatic basis for mental disease. In viewing the narratives and traces within archival records, we can find ourselves fixating upon an object that appears to encapsulate a programme of research and to tell a complete and coherent story. The neat piece of paper containing a pulse tracing – as the apparent end point of an experimental endeavour and often the only trace of the experiment that remains for the historian – can suggest that the production of the artefact itself was the aim of contemporary researchers, leading to a rather reductive view of historical medical or scientific practice. Rather, as this article has attempted to show, that artefact was one which emerged from a series of practices, affected by a variety of actors; it represented a visual narrative whose value and accuracy were the subject of continued debate. As a way of ‘knowing’ mental disease, the sphygmograph was one of many that took the body as its starting point. In charting the use of this instrument, we glimpse the many things that it was thought to materialize (e.g. heightened arterial tension, the presence of mania or melancholia) as well as the challenges and opportunities that it presented: the efforts of doctors to address patient experiences (the need to put them at ease), the bodily anomalies indicated by sphygmographic tracings (vessel spasm), and subsequent treatment options (Calabar bean). In examining the history of psychiatry in an era expressly concerned with the state of the body, the analysis of asylum practice is essential, offering new perspectives on scientific research and the place of medical technologies in the asylum. The often detailed and personalized accounts of sphygmography contrast with accounts of medical technology as a somewhat dehumanizing force that lessened the value placed on the subjective experiences of patients. In the cases discussed here, although an instrument may have offered a new way of ‘knowing’ mental disease, this did not necessarily mean that it eclipsed earlier perspectives and methods, nor that it was employed as a simple technological curiosity without reference to clinical intervention.

## Notes

1. A notable exception to this trend is Jacyna and Casper (2012), where psychiatric bodies of various kinds are demonstrated to be much more complex entities than the rather passive bodies of previous narratives.

2. West Yorkshire Archive Service, Wakefield (hereafter WYAS) C85/3/6/134 Male medical casebook M40: 437–440.

3. Bristol Record Office (hereafter BRO) 40513/C/2/4 Male casebook volume 13: 223.

4. WYAS C85/3/6/21 Female medical casebook F21: 565.

5. WYAS C85/3/6/134 Male medical casebook M40: 281–283.

6. WYAS C85/3/6/119 Male medical casebook M25: 29–32, 35–56.

7. WYAS C85/3/6/133 Male medical casebook M39: 319.

8. BRO 40513/C/3/7 Female casebook: 109.

9. BRO 40513/C/3/6 Female casebook: 35.

10. BRO 40513/C/3/7 Female casebook: 169.

11. WYAS C85/3/6/115 Male medical casebook M21: 351.

12. WYAS C85/1/12/3 Report of the Medical Superintendent, 26 January 1871: 25.

13. BRO 40513/C/3/7 Female casebook; 40513/C/3/6 Female casebook.

14. WYAS C85/3/6/122 Male medical casebook M28: 541–543.

15. BRO 40513/C/2/4 Male casebook volume 13: 206.

16. BRO 40513/C/2/4 Male casebook volume 13: 206.

17. WYAS C85/3/6/134 Male medical casebook M40: 453.
